# Case of Epileptic Asphyxia

**Published:** 1833-02

**Authors:** E. W. Duffin

**Affiliations:** 3, Foley place


### CASE OF EPILEPTIC ASPHYXIA.

*To the Editors of the London Medical and Physical Journal.*

GENTLEMEN: Should the following case appear to you of sufficient interest to merit publication, be so obliging as to give it a place in your valuable periodical.

Yours, sincerely,

E. W. DUFFIN.

3, *Foley place; 16th January*, 1833.

ON the evening of the 19th October last, I was requested to visit Mr. R., ætat. seventy-three years, of a sanguine temperament, slender and delicate form, and, for his age, a man of more than ordinary acuteness of intellect. I was informed that for the last four years he had been liable to fits, which recurred at irregular intervals, as often as three, four, or five times in the course of the year; that he had that morning, about eleven o'clock, been seized in the street, carried home, and cupped freely at the back of the neck; and that, since that time, he had experienced several more attacks, varying in duration from five to fifteen minutes each. On my entering his chamber, he was seized with a more than ordinarily severe paroxysm, which presented the following phenomena: At the moment of attack, he uttered a faint exclamation; his face flushed a little; his eyes became fixed; his breathing laborious, but not stertorous; and his whole frame slightly convulsed, more especially the muscles of the face and upper extremities. His pulse beat only twenty-eight in the minute, was free, soft, and easily compressed. In this condition he continued for about five minutes, his pulse becoming gradually slower and more feeble, until it beat only at intervals of twelve seconds, and then ceased altogether, at the heart as well as at the wrist simultaneously. His respiration, in like manner, became gradually less laborious, and was repeated at very long intervals, till it finally ceased. The colour that had remained in his cheeks now completely fled, the lighter parts of the skin assumed a leaden hue, and his lips a purple tint; the whole muscular system was relaxed, the lower jaw dropped, (for he was sitting supported in an easy chair,) the extremities became livid and cold, and I concluded he was dead.

As I was informed by his friends that he had repeatedly during the day, and on former occasions, exhibited similar phenomena and revived, I kept my finger applied to his pulse, and my hand over his heart, and requested an attendant to hold my watch before me, that I might ascertain how long animation would remain in a state of suspension. At the expiration of four minutes and a half, a single feeble pulsation was again felt, both at the heart and wrist, and the colour of his face began to return. In the space of six seconds, another pulsation was felt, and again after a lapse of four seconds: this third beat was much stronger and more decided in every respect than the two preceding ones. He then made an effort to breathe; and gradually from this time, in the space of about eight minutes, was perfectly restored, and his intellect became as clear as if nothing had happened to him.

For several days afterwards he was attacked, as often as ten times within the twenty-four hours, with fits of asphyxia similar to that I have just described, though only on one occasion of so long duration; and experienced in all, before recovery, forty-six paroxyms.

It is worthy of remark, that his pulse generally averaged twenty-eight beats in a minute during the intervals, and that afterwards, when in perfect health, it never exceeded thirty-three. For a few seconds before each fit, he generally complained of fulness and constriction of the head, and observed that, at the moment of seizure, a sudden determination of blood to the head, accompanied by a rushing noise, gave him acute pain, and was the cause of the exclamation he uttered at that time. Upon one occasion during his illness, he was very much distressed with spasm of the diaphragm, intercostal, and sterno-costalis muscles, which created difficulty of breathing to so great an extent, that I feared he would be suffocated.

Neither at this time, however, nor at any other, could I, by the most careful examination of his chest, detect any symptoms of disease of the heart: it always beat with the utmost regularity and tranquillity, and, if its slowness be excepted, naturally in every respect: but of this peculiarity he was aware, and informed me that it was natural to him, and had always been observed by every medical man that had attended him during his life. Nor did there exist any symptoms of organic disease of the brain: on the contrary, his mind, during the intervals and afterwards, was always clear, his memory good, and his judgment correct; and, unless for a moment or two before the accession of each fit, he never suffered headach nor other cerebral inconvenience.

From the history he gave me of certain dyspeptic symptoms, that had always apparently been the cause of his former attacks, combined with the existing foul and furred state of his tongue, loaded and obstinate bowels, and the insufferably offensive nature of the first discharges that were procured from them, I concluded that his attacks depended upon a surcharged and disordered state of the primæ viæ. Hence, as his pulse did not warrant further depletion, the treatment consisted of the administration of stimulating purgative clysters, repeated doses of calomel and compound extract of colocynth, and black draught; cold applications to the head, previously shaven; blisters to the nape of the neck; and, during the paroxysms, active friction over the whole body, and sinapisms to the feet. So soon as his tongue became clean, and the alvine evacuations had assumed a natural character, the fits ceased, and in ten days' time he was as well as he had ever been for years.

On the 20th of December following, he was again seized with another series of fits, for which, as his friends had seen him apparently so much worse and recover, they did not at first deem it necessary to send for medical aid, or pursue any active means to effect his restoration. In the course of the evening, however, observing him paralysed, I was requested to visit him; but it was too late; he died in a few hours afterwards.

It is to be regretted that permission could not be obtained to examine the body after death.

---

